# Targeting neutrophils extracellular traps (NETs) reduces multiple organ injury in a COVID-19 mouse model

**DOI:** 10.1186/s12931-023-02336-2

**Published:** 2023-03-02

**Authors:** Flavio P. Veras, Giovanni F. Gomes, Bruna M. S. Silva, Diego B. Caetité, Cicero J. L. R. Almeida, Camila Meirelles S. Silva, Ayda H. Schneider, Emily S. Corneo, Caio S. Bonilha, Sabrina S. Batah, Ronaldo Martins, Eurico Arruda, Alexandre T. Fabro, José C. Alves-Filho, Thiago M. Cunha, Fernando Q. Cunha

**Affiliations:** 1 Center of Research in Inflammatory Diseases (CRID), Ribeirão Preto Medical School, University of São Paulo, Av. Bandeirantes; Rua das Paineiras, Casa 3, Ribeirão Preto, São Paulo 14049-900 Brazil; 2 Department of Pharmacology, Ribeirão Preto Medical School, University of São Paulo, Ribeirão Preto, São Paulo Brazil; 3 Department of Pathology and Legal Medicine, Ribeirão Preto Medical School, University of São Paulo, Ribeirão Preto, São Paulo Brazil; 4 Laboratory of Experimental Pathophysiology, Graduate Program in Health Sciences, Health Sciences Unit, University of Southern Santa Catarina, Criciúma, Santa Catarina Brazil; 5 Virology Research Center, Ribeirão Preto Medical School, University of São Paulo, Ribeirão Preto, São Paulo Brazil; 6 Department of BioMolecular Sciences, School of Pharmaceutical Sciences of Ribeirão Preto, Universidade de São Paulo, Ribeirão Preto, SP Brazil

**Keywords:** Neutrophils, Neutrophil extracellular traps, Organ damage, COVID-19, SARS-CoV-2

## Abstract

**Background:**

COVID-19 is characterized by severe acute lung injury, which is associated with neutrophil infiltration and the release of neutrophil extracellular traps (NETs). COVID-19 treatment options are scarce. Previous work has shown an increase in NETs release in the lung and plasma of COVID-19 patients suggesting that drugs that prevent NETs formation or release could be potential therapeutic approaches for COVID-19 treatment.

**Methods:**

Here, we report the efficacy of NET-degrading DNase I treatment in a murine model of COVID-19. SARS-CoV-2-infected K18-hACE2 mice were performed for clinical sickness scores and lung pathology. Moreover, the levels of NETs were assessed and lung injuries were by histopathology and TUNEL assay. Finally, the injury in the heart and kidney was assessed by histopathology and biochemical-specific markers.

**Results:**

DNase I decreased detectable levels of NETs, improved clinical disease, and reduced lung, heart, and kidney injuries in SARS-CoV-2-infected K18-hACE2 mice. Furthermore, our findings indicate a potentially deleterious role for NETs lung tissue in vivo and lung epithelial (A549) cells in vitro*,* which might explain part of the pathophysiology of severe COVID-19. This deleterious effect was diminished by the treatment with DNase I.

**Conclusions:**

Together, our results support the role of NETs in COVID-19 immunopathology and highlight NETs disruption pharmacological approaches as a potential strategy to ameliorate COVID-19 clinical outcomes.

**Supplementary Information:**

The online version contains supplementary material available at 10.1186/s12931-023-02336-2.

## Background

Coronavirus disease 2019 (COVID-19) is an infection caused by the severe acute respiratory syndrome coronavirus 2 (SARS-CoV-2) [1, 2] and pulmonary-related symptoms are one of its hallmarks [3, 4]. Neutrophils have been described as indicators of the severity of respiratory symptoms and poor COVID-19 prognosis [5–7]. Neutrophil extracellular traps (NETs) are one of the most relevant effector mechanisms of neutrophils in inflammatory diseases, playing a central role in organ damage [8–10].

NETs are web-like structures of extracellular DNA fibers containing histones and granule-derived enzymes, such as myeloperoxidase (MPO), neutrophil elastase, and cathepsin G [11, 12]. The formation of NETs is known as NETosis, and it starts with neutrophil activation by pattern recognition receptors (TLRs, e.g.) or chemokines. The process is followed by ROS production and calcium mobilization, which leads to the activation of protein arginine deiminase 4 (PAD-4) [13]. The activation of the neutrophil elastase also plays a role in NETs production in inflammatory responses [14–17].

Elevated levels of NETs are found in the blood, thrombi, and lungs of patients with severe COVID-19, suggesting that neutrophils and NETs may play an important role in the pathophysiology of COVID-19 [8–10].

Drug repurposing is a key strategy to accelerate the discovery of new effective treatments for COVID-19 [18]. In this context, as the literature shows evidence of the role of NETs in COVID-19, DNase I, an FDA-approved drug that degrades NETs could be proposed as a potential new candidate for COVID-19 treatment [19–21]. Dornase alfa (recombinant human DNase I) is broadly used to improve lung function of patients with Cystic Fibrosis [19–21]. This drug significantly reduces mucus viscosity by degrading extracellular DNA in the airways [19–21]. Thus, we propose DNase I as a therapeutic agent to reduce NETs in COVID-19, potentially improving clinical outcomes, pulmonary function, and, consequently, the prognosis of the disease.

Here, we demonstrate that DNase I treatment decreases the concentration of NETs in the plasma and lungs of SARS-CoV-2-infected mice and ameliorates experimental COVID-19. These findings highlight the importance of NETs inhibitors as a potential therapeutic approach for COVID-19 treatment.

## Methods

### K18-hACE2 mice

K18-hACE2 humanized mice (B6·Cg-Tg(K18-ACE2)2Prlmn/J) were obtained from The Jackson Laboratory and were bred in the *Centro de Criação de Animais Especiais* (Ribeirão Preto Medical School/University of São Paulo). This mouse strain has been previously used as the model for SARS-CoV-2-induced disease and it presents signs of diseases, and biochemical and lung pathological changes compatible with the human disease [22]. Mice had access to water and food ad libitum. The manipulation of these animals was performed in Biosafety Levels 3 (BSL3) facility and the study was approved by the Ethics Committee on the Use of Animals of the Ribeirão Preto Medical School, University of São Paulo (#066/2020).

### DNase I treatment in SARS-CoV-2 experimental infection

Male K18-hACE2 mice, aged 8 weeks, were infected with 2 × 10^4^ PFU of SARS-CoV-2 (in 40 µL) by the intranasal route. Uninfected mice (n = 5) were given an equal volume of PBS through the same route. On the day of infection, 1 h before virus inoculation, animals were treated with DNase I (10 mg/kg, s.c., Pulmozyme, Roche) (n = 6) or vehicle (PBS, s.c.) (n = 6). DNase I was also given once a day until 5 days post-infection. Body weight was evaluated on the baseline and all days post-infection. The right lung was collected, harvested, and homogenized in PBS with steel glass beads. The homogenate was added to TRIzol reagent (1:1), for posterior viral titration via RT-qPCR, or to lysis buffer (1:1), for ELISA assay, and stored at − 70 °C. The left lung was collected in paraformaldehyde (PFA, 4%) for posterior histological assessment.

### H&E staining and lung pathology evaluation

Five μm lung, heart, and kidney slices were submitted to Hematoxylin and Eosin staining. A total of 10 photomicrographs in 40X magnification per animal were randomly obtained using a microscope ScanScope (Olympus) and Leica. Morphometric analysis was performed by the protocol established by the American Thoracic Society and European Thoracic Society (ATS/ERS) [23].

### NETs quantification

Plasma or homogenate from the lung was incubated overnight in a plate pre-coated with anti-MPO antibody (Thermo Fisher Scientific; cat. PA5-16672) at 4 °C. The plate was washed with PBS-T (Phosphate-Buffered Saline with Tween 20). Next, samples were incubated overnight at 4 °C. Finally, the plate with samples was washed and over MPO-bound DNA was quantified using the Quant-iT PicoGreen kit (Invitrogen; cat. P11496).

### Cytokines and chemokines levels

Lung homogenate was added to the RIPA buffer solution (Sigma-Aldrich, cat. R0278) and centrifuged at 10,000 g at 4 °C for 10 min. The supernatant was collected. The ELISA method was performed to detect the concentration of cytokines and chemokines using kits from R&D Systems (DuoSet), according to the manufacturer’s instructions. The following targets were evaluated: TNF-α, IL-6, IL-10, CXCL1, CCL2, and CCL4.

### Immunofluorescence and confocal microscopy

Lungs were harvested and fixed with PFA 4%. After dehydration and paraffin embedding, 5 μm sections were prepared. The slides were deparaffinized and rehydrated by immersing the through Xylene and 100% Ethanol 90% for 15 min, in each solution. Antigen retrieval was performed with 1.0 mM EDTA, 10 mM Trizma-base, pH 9·0 at 95 °C for 30 min. Later, endogenous peroxidase activity was quenched by incubation of the slides in 5% H_2_O_2_ in methanol for 15 min. After blocking with IHC Select Blocking Reagent (Millipore, cat. 20773-M) for 2 h at room temperature (RT), the following primary antibodies were incubated overnight at 4 °C: goat polyclonal anti-myeloperoxidase (anti-MPO, R&D Systems, cat. AF3667, 1:100) and rabbit polyclonal, anti-histone H3 (H3Cit; Abcam; cat. ab5103; 1:100). The slides were then washed with TBS-T (Tris-Buffered Saline with Tween 20) and incubated with secondary antibodies donkey anti-goat IgG Alexa Fluor 488 (Abcam, cat. ab150129) and alpaca anti-rabbit IgG AlexaFluor 594 (Jackson ImmunoReseacher; Cat. 611-585-215; 1:1000). Autofluorescence was quenched using the TrueVIEW Autofluorescence Quenching Kit (Vector Laboratories, cat. SP-8400-15). Slides were then mounted using Vectashield Antifade Mounting Medium with DAPI (Vector Laboratories, Cat# H-1200-10). Images were acquired by Axio Observer combined with LSM 780 confocal microscope (Carl Zeiss) at 63X magnification at the same setup (zoom, laser rate) and tile-scanned at 4 fields/image. Images were analyzed with Fiji by Image J.

### Measurement of organ damage biomarkers

Renal dysfunction was assessed by the levels of blood creatinine, and creatine kinase-MB was used as an index of cardiac lesions. The determinations were performed using a commercial kit (Bioclin).

### Neutrophils isolation and NETs purification

Peripheral blood samples were collected from healthy controls by venipuncture and the neutrophil population was isolated by Percoll density gradient (GE Healthcare; cat. 17-5445-01). Isolated neutrophils (1.5 × 10^7^ cells) were stimulated with 50 nM of PMA (Sigma-Aldrich; cat. P8139) for 4 h at 37 °C. The medium containing NETs was centrifuged at 450 g to remove cellular debris for 10 min, and NETs-containing supernatants were collected and centrifuged at 18,000 g for 20 min. Supernatants were removed, and DNA pellets were resuspended in PBS. NETs were then quantified with a GeneQuant (Amersham Biosciences Corporation).

### Apoptosis assay

Lung tissue was harvested for detection of apoptotic cells in situ with Click-iT Plus TUNEL Assay Alexa Fluor 488, according to the manufacturer’s instructions (Thermo Fisher Scientific; cat. C10617). Human alveolar basal epithelial A549 cells (5 × 10^4^) were maintained in DMEM and cultured with purified NETs (10 ng/ml) pretreated, or not, with DNase I (0·5 mg/ml; Pulmozyme, Roche). The cultures were then incubated for 24 h at 37 °C. Viability was determined by flow cytometric analysis of Annexin V staining.

### Flow cytometry

Lung tissue was harvested and digested with type 2 collagenase to acquire cell suspensions. Cells were then stained with Fixable Viability Dye eFluor 780 (eBioscience; cat. 65-0865-14; 1:3000) and monoclonal antibodies specific for CD45 (BioLegend; clone 30-F11; cat. 103138; 1:200), CD11b (BD Biosciences; clone M1/70; cat. 553311) and Ly6G (Biolegend; clone 1A8; cat. 127606) for 30 min at 4 °C. A549 cells (1 × 10^5^) were stained with FITC ApoScreen Annexin V Apoptosis Kit (SouthernBiotech; cat. 10010-02), according to the manufacturer’s instructions. Data were collected on a FACSVerse (BD Biosciences) and analyzed with FlowJo (TreeStar).

### Statistical analysis

Statistical significance was determined by either two-tailed unpaired Student t-test, and by one-way or two-way ANOVA followed by Bonferroni’s post hoc test. P < 0·05 was considered statistically significant. Statistical analyses and graph plots were performed and built using GraphPad Prism 9·3·1 software.

## Results

### DNase I reduces clinical outcomes in an experimental model of COVID-19

**Fig. 1:**
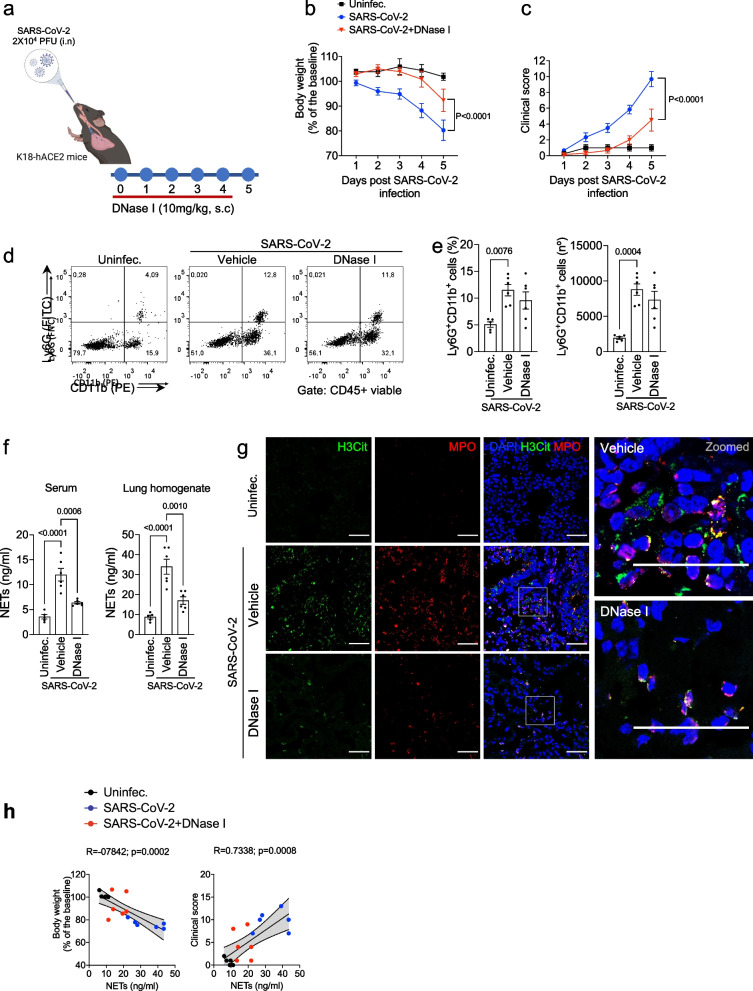
DNase I reduces clinical outcomes in a COVID-19 experimental model. **a** K18-hACE2 mice (n = 6) were intranasally (i.n) inoculated with SARS-CoV-2 (2 × 10^4^ PFU) and treated with DNase I (10 mg/kg, s.c) for 5 days. Created with Biorender.com. The uninfected group was used as negative control (n = 5). Weight loss and clinical scores were analyzed **(b, c)**. Representative dot plots **(d)**, frequency, and total number **(e)** of Ly6G + CD11b + cells were assessed by flow cytometry. **f** NETs quantification by MPO-DNA PicoGreen assay from serum and lung tissue homogenate. **g** Immunofluorescence analysis of H3Cit (green) and MPO (red) expression from the lung of SARS-CoV-2-infected mice and treated with DNase I. DAPI (blue) was used for nuclei staining. The scale bar indicates 50 µm. **h** Pearson’s correlation between body weight or clinical score and NETs concentration in lung homogenate. Data are representative of at least two independent experiments and are shown as mean ± SEM. P values were determined by two-way **(b, c)** and one-way ANOVA **(e, f)**

To investigate the role of NETs in COVID-19 pathophysiology we used K18-hACE2 mouse intranasally infected with SARS-CoV-2; [22, 24]. Infected mice were treated with saline or DNAse I (10 mg/kg; s.c.) administered 1 h before virus infection and once daily up to 4 days after infection (Fig. 1a). We evaluated body weight loss from baseline measure (basal) and clinical scores, as read out of disease progression (Additional file 1: Table S1). Our data showed that DNase I treatment attenuates both body weight loss and clinical score caused by SARS-CoV-2 infection compared to vehicle-treated mice (Fig. 1b, c).

We observed an increase in NETs concentration (Fig. 1f, g) and the number of neutrophils in the lung of infected mice upon induction of the COVID-19 model (Fig. 1d, e). These data corroborate some findings published by either our group or other authors, showing the presence of systemic levels of NETs, as well as localized in pulmonary tissue from COVID-19 patients [8–10].

DNase I treatment did not alter the number of neutrophils that infiltrated into the lung tissue of SARS-CoV-2 infected mice. The FACS analysis showed that the frequency and absolute numbers of neutrophils (Ly6G + CD11b + cells; Fig. 1d, e) were not altered by the treatment. Similar results were observed when analyzing CD45 + cell populations (Additional file 1: Fig. S1). However, we demonstrate that DNAse I treatment of SARS-CoV-2-infected mice resulted in a substantial reduction of NETs production that associates with a lower disease score (Fig. 1h). These data suggest that NETs play a crucial role in SARS-CoV-2 infection.

### Inhibition of NETs ameliorates lung pathology in SARS-CoV-2-infected mice

Lung inflammation is the primary cause of life-threatening respiratory disorders in critical and severe forms of COVID-19 [3, 4, 25]. NETs have been identified in the lung tissue of SARS-CoV-2 infected patients and lung injury experimental animal models [8, 9, 26]. Thus, we next investigated the effect of NETs degradation by DNase I treatment of the COVID-19 mouse model.

**Fig. 2:**
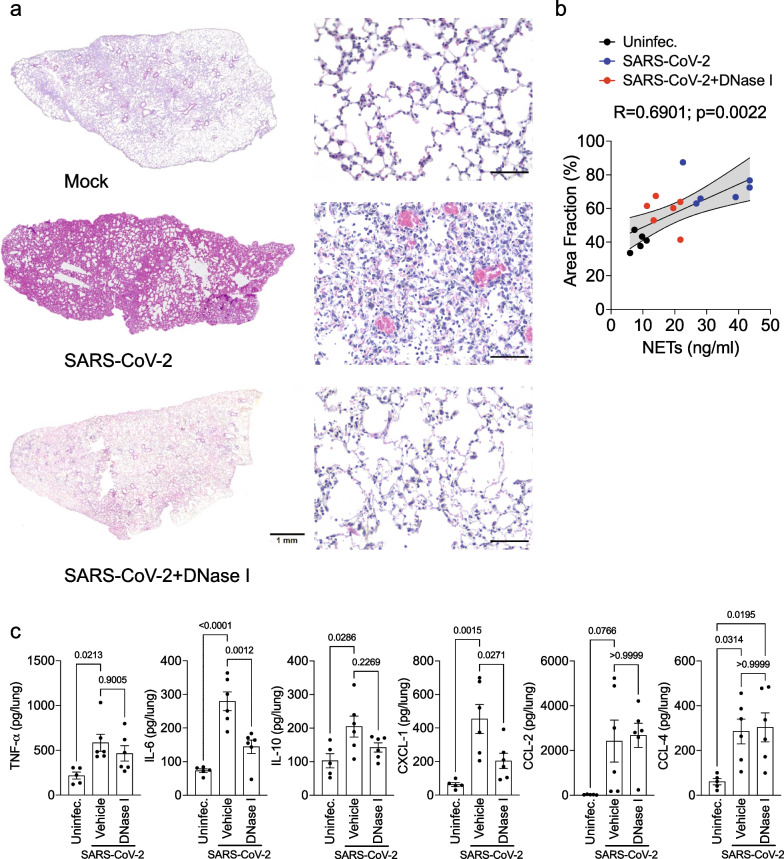
Pharmacological degradation of NETs ameliorates lung pathology upon SARS-CoV-2 infection in vivo. K18-hACE2 mice (n = 6) were intranasally (i.n) inoculated with SARS-CoV-2 (2 × 10^4^ PFU) and treated with DNase I (10 mg/kg, s.c) for 5 days. **a** Representative H&E staining from the lung of mock or SARS-CoV-2-infected mice and treated with DNase I. **b** Pearson correlation between the area fraction and NETs concentration in lung homogenate. The total septal area and the total lung area were quantified using the Pro Plus 7 software (Media Cybernetics, Inc., MD, USA). The ratio between total septal area and the total lung area was expressed as area fraction (%). **c** an ELISA assay was performed to detect TNF-α, IL-6, IL-10, CXCL-1, CCL-2, and CCL-4 levels from the lung. Data are representative of two independent experiments and are shown as mean ± SEM. P values were determined by one-way ANOVA

We noticed an extensive injury in the lung tissue of K18-hACE2-infected mice with interstitial leukocyte infiltration. The alveolar units showed architectural distortion compromising the alveolar-capillary barrier. DNase I treatment was able to prevent lung damage caused by SARS-CoV-2 infection (Fig. 2a). Moreover, analyzing the area fraction, as a score of septal thickness, DNase I-treatment reduced the area fraction in SARS-CoV-2-infected mice and was associated with a lower concentration of NETs released (Fig. 2b). The reduction in lung pathology was associated with a reduction of pro-inflammatory cytokines/chemokines, especially IL-6 and CXCL-1 in the lung tissue of mice treated with DNase I (Fig. 2c). These results indicate that pharmacological inhibition of NETs could be a novel approach to inhibit SARS-CoV-2-induced lung pathology.

### DNase I attenuates extrapulmonary injuries in the COVID-19 mouse model

**Fig. 3:**
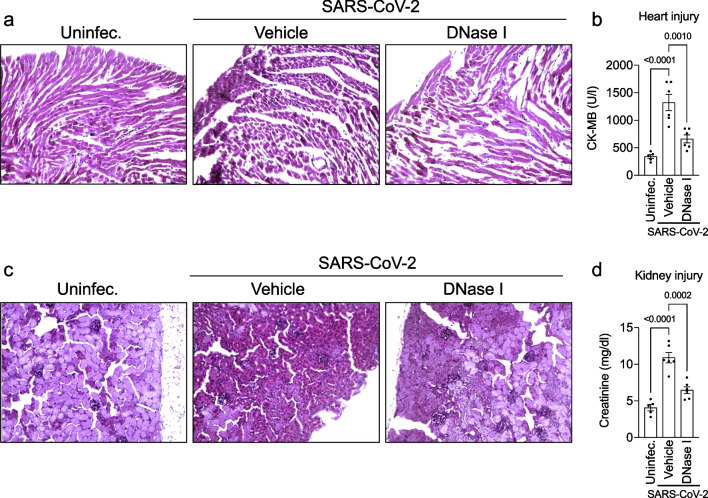
NETs degradation attenuates heart and kidney failure in a COVID-19 experimental model. K18-hACE2 mice (n = 6) were intranasally (i.n) inoculated with SARS-CoV-2 (2 × 10^4^ PFU) and treated with DNase I (10 mg/kg, s.c) for 5 days. Representative H&E staining from the heart **(a)** and kidney **(c)** of mock, SARS-CoV-2-infected mice and treated with DNase I. Serum levels of creatine kinase-MB (CK-MB) **(b)** and creatinine **(d)** determined by colorimetric assays. Data are representative of two independent experiments and are shown as mean ± SEM. P values were determined by one-way ANOVA

COVID-19 is a systemic viral disease that can affect other vital organs besides the lungs, such as the heart and kidney [27–30]. To investigate this context, we harvested the heart and kidneys of animals 5 days post-infection and evaluated whether NETs inhibition could change this phenotype. We observed that the heart of mice infected with SARS-CoV-2 showed pathological changes in cardiac tissue with diffuse and sparse cardiac inflammatory cell infiltration and perivascular injury (Fig. 3a). Moreover, after treatment, there is a clear reduction of inflammatory cells in heart tissue, leaving “injured” (fine) fibers and interstitial edema, similar to what is seen in autoimmune myocarditis. In addition, plasma Creatine Kinase MB (CK-MB) concentration in the infected group was higher than in control mice (Fig. 3b). Interestingly, DNase I treatment attenuated the pathology and CK-MB concentration.

Finally, we aimed to investigate whether SARS-CoV-2 infection induces kidney damage in K18-hACE2 mice. Consistent with lung and heart pathological changes, we found the presence of ischemic tubulointerstitial nephritis in the COVID-19 model, mimicking acute tubular necrosis with cellular glomerulitis (Fig. 3c). Furthermore, the levels of creatinine in the blood of infected mice were higher in comparison with uninfected animals (Fig. 3d). As expected, DNase I treatment reduced kidney injury (Fig. 3c, d). Collectively, these findings indicate that pharmacological inhibition of NETs with DNase I prevents multi-organ dysfunction in COVID-19.

### DNase I prevents NETs-induced apoptosis of lung tissue in SARS-CoV-2-infected mice

To understand the possible role of NETs in the pathophysiology of COVID-19, we explored the hypothesis that NETs could be involved in lung damage accompanied by the disease, as previously described [9, 31].

**Fig. 4:**
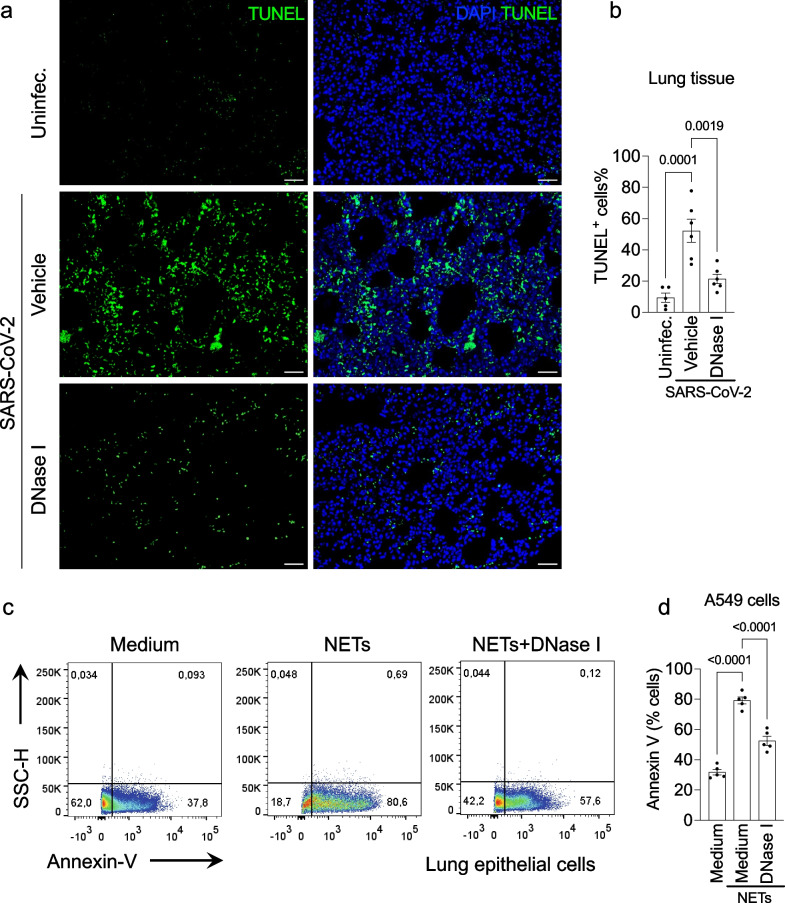
DNase I prevents apoptosis of lung tissue and lung epithelial cells. K18-hACE2 mice (n = 6) were intranasally (i.n) inoculated with SARS-CoV-2 (2 × 10^4^ PFU) and treated with DNase I (10 mg/kg, s.c) for 5 days. **a** TUNEL staining (green) for detection of apoptotic cells in situ from lung tissue of mice. **b** Percentage of TUNEL-positive cells in lung tissue. NETs were purified from healthy neutrophils and stimulated with PMA (50 nM) for 4 h at 37 °C **c** Representative dot plots of FACS analysis of Annexin V + A549 cells incubated with purified NETs (10 ng/ml) pretreated, or not, with DNase I (0.5 mg/ml) for 24 h at 37 °C. **d** Frequency of Annexin V + A549. Data are representative of two independent experiments and are shown as mean ± SEM. P values were determined by one-way ANOVA followed by Bonferroni’s post hoc test

To this end, lung analysis of k18-hACE2 mice infected with SARS-CoV-2 reveals a massive presence of apoptotic cells after 5 days of infection. DNase I treatment was able to prevent lung apoptosis caused by the COVID-19 mouse model (Fig. 4a). Our analysis showed a reduction of approximately 60% in TUNEL-positive cells after NETs degradation (Fig. 4b).

In our next step, purified NETs from healthy human neutrophils were incubated with A549 cells, a human alveolar basal epithelial cell, and cell viability was determined (annexin V + cells) by flow cytometry**.**

We found that exposure of A549 cells to purified NETs significantly increased the percentage of apoptotic cells in comparison with untreated cells. Importantly, the pretreatment of purified NETs with DNase I prevented NETs-induced epithelial (Fig. 4c, d) apoptosis to similar levels observed in untreated cells. Taken together, these results are evidence that DNase I exhibits a protective effect on NETs deleterious functions in lung tissue and could be a potential strategy to block organ injury during COVID-19.

## Discussion

While the number of patients with COVID-19 is growing worldwide, there is no effective treatment for the disease [32, 33]. Thus, the understanding of the mechanisms by which the hosts deal with the SARS-CoV-2 virus could allow the development of new therapeutic strategies aiming to prevent tissue injuries triggered by the infection. Here, we report that in a COVID-19 mouse model, NETs are released systemically and in higher concentrations in the lungs of K18-hACE2 mice. Moreover, DNase I treatment reduced multi-organ lesions and improved outcomes associated with NETs released.

The increase in the number of circulating neutrophils is an indicator of a worse outcome of COVID-19 [34]. In 2004, Brinkmann et al. described, for the first time, that NETs are released by neutrophils, and work as a microbicidal mediator [11]. However, following ability, NETs mediate lesions observed in several inflammatory diseases, including rheumatoid arthritis, lupus, diabetes, and sepsis [8, 10, 35–39]. Inhibition of NETs production prevented lung, heart, and liver lesions observed in experimental sepsis [38–40].

The SARS-CoV-2 infection affects the lungs and multiple organs, occasionally causing death. Besides immunizations, strategies to prevent organ dysfunction in patients with COVID-19 are of main importance. Emerging evidence implicates that NET formation plays a pivotal role in the pathophysiology of inflammation, coagulopathy, organ damage, and immunothrombosis that characterize severe cases of COVID-19 [8, 9].

Several stimuli trigger NETs release, including pathogen-associated molecular patterns (PAMPs), damage-associated molecular patterns (DAMPs), and inflammatory mediators, studies demonstrated that NETs have a dual biological role. Besides their microbicide such as cytokines and chemokines [41–43]. Our group has previously demonstrated that SARS-CoV-2 can directly infect human neutrophils and is key to triggering NETs production. The first step in neutrophil infection by SARS-CoV-2 is the interaction of the virus with ACE2 and TMPRSS2 expressed on the surface of human neutrophils [8]. It is possible to speculate the participation of the cytokines and chemokines released by host cells as activators [44, 45] of NETs production by the neutrophils in the K18-hACE2 model; however, this deserves future investigation.

In humans, the response to SARS-CoV-2 infection is comprised of cytokines and chemokines production [46]. Here, we show that SARS-CoV-2 infection of K18-hACE2 mice elicits a measurable systemic pro-inflammatory cytokine response which is significantly increased at 5 DPI and characterized by an increase in TNF-α, IL-6, and IL-10, and encompasses upregulation of cell-recruiting chemokines CXCL-1, CCL-2, and CCL-4. Importantly, increased levels of TNF-α and IL-6 are associated with the severity of disease in COVID-19 patients [47]. In addition, cytokine levels are also reported to be indicative of extrapulmonary multiple-organ failure [48, 49]. Interestingly, DNase I could prevent systemic inflammation in some COVID-19 patients, including the reduction of pro-inflammatory cytokines TNF-α and IL-6 [50, 51]. This needs to be further investigated to clarify if our observation suggests a differently modulated immune response and pathogenesis by NETs levels.

NETs play a paradoxical role. Once released, they play an important microbicidal role due to their toxic content, assisting the capture and inactivation of different types of pathogens, including viruses [11, 13, 41, 43, 52]. However, in excess, these traps can also cause significant tissue damage, as seen in rheumatoid arthritis [35], diabetes [37], prothrombotic events, and sepsis [39, 40]. Our group and others have demonstrated a significant increase in the concentration of NETs in the plasma and also in the lung tissues of patients with COVID-19 [8, 10]. Thus, combining antiviral with the control of NETs might be a strategic option to treat short-living virus-caused pathologies, especially COVID-19. Although we demonstrated the presence of NETs, cell-free DNA can exert a role during COVID-19 as demonstrated by some authors in patients’ samples. However, the massive production of cell-free DNA is from NETs as recently demonstrated [53].

The literature describes that NETs can present direct cytotoxic effects on different mammalian cell types, including epithelial and endothelial cells, inducing apoptosis or necrosis [12, 54]. Moreover, NETs could also activate different PRR receptors, such as toll-like receptor (TLR)-4 and 9, which mediate the release of inflammatory mediators; in turn, amplifying the direct effects of NETs [35]. In this context, during COVID-19, apoptosis of lung epithelial was previously observed. These events are capable of compromising lung function, worsening the severity of the disease [3, 4]. Considering these findings, in the present study, we observed that DNase I prevented apoptosis in lung tissue from SARS-CoV-2-infected mice. In accordance observed in the COVID-19 model, isolated NETs from the culture of PMA-stimulated human neutrophils induced in vitro apoptosis of A549 epithelial cells with reversal homeostasis in presence of DNase I. A549 is a great tool for mimicking the lung inflammatory environment and was used as a model to investigate NET-induced apoptosis of lung cells, as stated above. In future work, we intend to investigate the molecular mechanisms of NET-induced apoptosis in lung cells*.*

Extending this finding to COVID-19 disease, it is possible to suggest that the reduction of viability of the lung cells is a consequence of the local production of NETs. In this line, we observed the presence of neutrophils releasing NETs, as well as a high concentration of NETs in the lung of SARS-CoV-2-infected mice. It is reported that DNase reduces NETosis in the plasma of SARS-CoV-2-infected patients, alleviates systemic inflammation, and attenuates mortality in a septic mouse model [51].

In patients with severe COVID-19 pneumonia, treatment with DNase I, in a randomized clinical trial, resulted in significant anti-inflammatory effects and reduced markers of immune pathology [55]. In another clinical trial, treatment with DNase I was associated with improved oxygenation and decreased NETs lung fluid [21]. These data indicate that the degradation of NETs or NET-associated structures by inhaled DNase I can be beneficial in the context of pulmonary diseases. The effectiveness of DNase I in patients with acute respiratory infection and inflammation corroborates our experimental findings. Some authors described that NETs are found in inflamed lungs of hamsters inoculated with SARS-CoV-2 that could mediate immunothrombosis and lung injury [26]. Indeed, the experimental inhibition of NET formation in SARS-CoV-2-infected K18-hACE2 mice could attenuate the development of signs of disease, and act in the reduction of lung pathology and cytokine storm [38, 56]. In summary, our findings support NETs as a target for improving COVID-19 clinical outcomes.

## Conclusions

We observed the presence of neutrophils releasing NETs in the lung tissue of infected mice, detected in the alveolar space. Disease progression was prevented with DNase I treatment in vivo. This observation is tightly correlated with the development of lung injury, suggesting that strategies to reduce NETs levels could have favorable effects on recovering lung function.

Together, our findings demonstrate the potentially deleterious role of NETs during COVID-19 and support the use of inhibitors of NETs, such as DNase I as a strategy to ameliorate multi-organ damage during COVID-19.

## Supplementary Information

**Additional file 1. Figure S1.** DNase I treatment does not alter leukocyte accumulation in the lungs of SARS-CoV-2-infected mice. K18-hACE2 mice (n=6) were intranasally (i.n) inoculated with SARS-CoV-2 (2x10^4^ PFU) and treated with DNase I (10mg/kg, s.c) for 5 days. (a) Doublets, debris, and dead cells were first excluded. Leukocytes were identified as CD45+ events among viable cells. (b) Flow cytometry analyses of CD45+ living cells from the lung of K18-hACE2 infected mice treated or not with DNase I. Uninfected was used as control. **Table S1.** Criteria of clinical sickness score.

## Data Availability

All of the data generated or analyzed during this study are included in this article and the additional information. All data supporting the findings of this study are also available upon reasonable request from the corresponding authors [F.Q.C. or F.P.V.].
