# Supplementary material for: Targeting neutrophils extracellular traps (NETs) reduces multiple organ injury in a COVID-19 mouse model
Source: Respir Res. 2023 Mar 2;24:66. doi: 10.1186/s12931-023-02336-2 (PMC9978286; doi:10.1186/s12931-023-02336-2)
Supplement: Supplementary file 1 — Additional file 1. Figure S1. DNase I treatment does not alter leukocyte accumulation in the lungs of SARS-CoV-2-infected mice. K18-hACE2 mice (n=6) were intranasally (i.n) inoculated with SARS-CoV-2 (2x10^4^ PFU) and treated with DNase I (10mg/kg, s.c) for 5 days. (a) Doublets, debris, and dead cells were first excluded. Leukocytes were identified as CD45+ events among viable cells. (b) Flow cytometry analyses of CD45+ living cells from the lung of K18-hACE2 infected mice treated or not with DNase I. Uninfected was used as control. Table S1. Criteria of clinical sickness score. [file 12931_2023_2336_MOESM1_ESM.docx]

**Supplementary material**

**Figure S1 – DNase I treatment does not alter leukocyte accumulation in the lungs of SARS-CoV-2-infected mice.** K18-hACE2 mice (n=6) were intranasally (i.n) inoculated with SARS-CoV-2 (2x10^4^ PFU) and treated with DNase I (10mg/kg, s.c) for 5 days. **(a)** Doublets, debris, and dead cells were first excluded. Leukocytes were identified as CD45+ events among viable cells. **(b)** Flow cytometry analyses of CD45+ living cells from the lung of K18-hACE2 infected mice treated or not with DNase I. Uninfected was used as control. Data are representative of two independent experiments and are shown as mean ± SEM. P values were determined by one-way ANOVA followed by Bonferroni’s post hoc test.
